# The Study of Manufacturing Thermal Insulation Materials Based on Inorganic Polymers under Microwave Exposure

**DOI:** 10.3390/polym14153202

**Published:** 2022-08-05

**Authors:** Tatyana Rymar, Halyna Tatarchenko, Oleksij Fomin, Václav Píštěk, Pavel Kučera, Martin Beran, Oleksij Burlutskyy

**Affiliations:** 1 Department of Chemical Engineering and Ecology, Volodymyr Dahl East Ukrainian National University, 59a Tsentralnyi Prospect, 93400 Severodonetsk, Ukraine; 2 Department of Construction, Urbanism and Spatial Planning, Volodymyr Dahl East Ukrainian National University, Central Avenue 59b, 93400 Severodonetsk, Ukraine; 3 Department of Cars and Carriage Facilities, State University of Infrastructure and Technologies, Kyrylivska Street, 9, 04071 Kyiv, Ukraine; 4 Institute of Automotive Engineering, Brno University of Technology, Technická 2896/2, 616-69 Brno, Czech Republic; 5 Department of Mechanics and Machine Design, Ukrainian State University of Railway Transport, Feuerbach Square, 7, 61050 Kharkiv, Ukraine

**Keywords:** microwave technology, composite thermal insulation material, liquid glass, properties, structure, microwave heating, convective heating

## Abstract

The work is devoted to the creation of an energy-saving microwave technology of composite materials for thermal insulation based on an inorganic polymer—liquid glass—and the establishment of the formation patterns of their structure and properties, depending on the parameters of microwave radiation. Due to volumetric heating and the mechanism of "non-thermal" action of microwave radiation on processed objects, the duration of their heating is significantly reduced, and the performance properties of products are improved due to the modification of the structure of the liquid glass matrix under the influence of its irradiation with this type of energy. In the course of the research, the relationship between the processing conditions and the obtained properties of heat-insulating materials was established. The prospects of using the microwave electromagnetic field in the production of new building materials, expanding their range, and improving quality and competitiveness are shown, which is a priority for the modernization and innovative development of the construction industry.

## 1. Introduction

**Figure 1:**
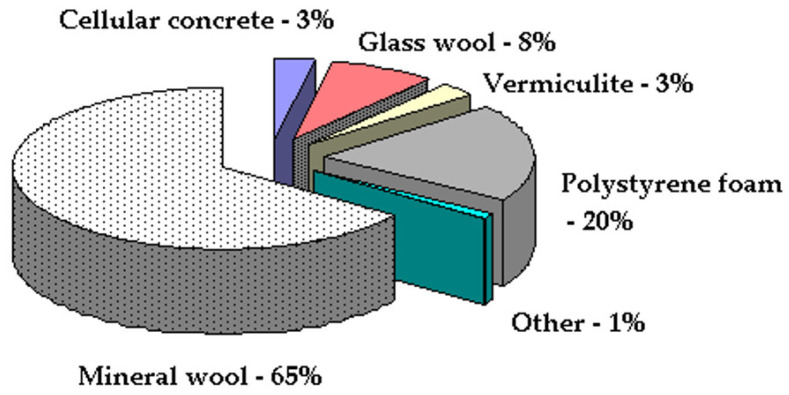
World market of insulation materials.

The world market of insulation materials is characterized by the dominance of inorganic fibrous materials; in particular, mineral wool, which accounts for 65% of the market, and organic foam materials, namely, extruded polystyrene and polyurethane, accounting for about 21% (Figure 1) [1].

Mineral wool insulation is cheaper than organic and is classified as a non-combustible product. In addition, it can withstand very high temperatures (500–750 °C) without losing its thermophysical properties. The two main disadvantages of using fibrous mineral wool are the low resistance to vapor diffusion that leads to an increase in thermal conductivity when vapor is absorbed, and the potential hazardous effects on human health. Mineral wool can cause irritation to the skin, eyes, and respiratory system; therefore, precautions are necessary during its manufacturing and processing [2]. In addition, this material has an increased deformation during operation, which can lead to an increase in its thermal conductivity coefficient over time.

The choice of materials for thermal insulation is determined primarily by the nature of the thermal protection object, the feasibility of its protection method, and the convenience of using materials during work. The thermal insulation of power equipment and pipelines requires thermal insulation materials to be incombustible and withstand high temperatures. Therefore, materials based on organic polymers cannot be used for this purpose, since their structure is destroyed, which leads to heat losses. Thermal insulation materials based on an inorganic polymer, i.e., liquid glass, fully meet these requirements. They are characterized by high environmental friendliness due to the absence of material depolymerization and the release of toxic substances into the atmosphere; heat resistance (operating temperature is up to 600 °C); non-combustibility; and biostability with a guaranteed service life of at least 50 years.

The main limiting factor in manufacturing these materials is the difficulty of obtaining thermal insulation products during the thermal swelling of liquid glass binders in furnaces with traditional convective heating. This is because of the slow heating of the inner layers of the liquid glass composition due to the low thermal conductivity of the swollen outer layer. This made it necessary to search for a new alternative technology for manufacturing thermal insulation products based on liquid glass, which could be used for the thermal insulation of high-temperature equipment including pipelines. This problem is proposed to be solved by using relatively new microwave technology. The advantage of microwave radiation is the volumetric heating from the inside of the material being processed, which makes it possible to obtain a volumetrically monolithic material by simultaneously swelling liquid glass granules and the binder, which is also swollen. The use of microwave radiation during the swelling and curing of these materials reduces the process duration and temperature, as well as improving the performance properties of products by modifying the structure of the liquid glass matrix under exposure to this type of energy.

The experience of using microwave radiation in the field of polymeric materials demonstrates a useful technological impact from the short-term exposure of polymers to the microwave electromagnetic field. Since the beginning of the 2000s, at Saratov State Technical University, the scientists of Yu.S. Arkhangelsk’s scientific school have been conducting research in the field of the non-thermal effect of the microwave electromagnetic field on thermally reactive and thermoplastic polymeric materials [3].

Kalganova S.G. and Arkhangelsky Yu.S. together with Sleptsova S.K. and Lavrentiev V.A. have studied the non-thermal effect of the microwave electromagnetic field on polymers [4,5,6,7,8]. Works [5,6] studied the curing mechanism of an epoxy compound, which consists in changing its topological structure, characterizing the distribution of globules and their frequency. Microwave radiation increases the energy of the chaotic thermal motion of monomers, thus, promoting their intense polymerization and the creation of cross-links. The low molecular weight fraction becomes less defective with a stronger cross-linked structure. A decrease in the structure defectiveness of the low molecular weight fraction and an increase in the size of densely reticulated globules in the structure of the cured epoxy compound leads to the modification of its properties. Microwave processing increases the tensile strength of the compound by 3–4 times, heat resistance by 1.4–1.6 times compared to the compound cured in the air under natural conditions, and intensifies its curing by tens of times (5–70 times) for different modes of microwave processing.

Works [7,8] studied the non-thermal microwave effect modes on the properties of fibrous polycaproamide and revealed that the specific breaking load of the fibre increases by 12–15%, and the relative elongation at break decreases by 20–25% compared to the initial polycaproamide sample. The effect is observed during short-term microwave exposure for 5–10 s when the temperature of the object remains constant during processing, which indicates the non-thermal nature of the microwave currents.

In 2009, in the Sterlitamak branch of Ufa State Petroleum Technological University, under the guidance of Professor Shulaev N.S., there began the studies of the electromagnetic field effects of the microwave range on the properties of polymeric insulation materials. The study idea was to establish the possibility of using microwave electromagnetic radiation to purposefully change the structure of polymers and improve their physical, mechanical, and performance properties [9,10,11].

Thus, the work considers the advantages of using electromagnetic radiation of the microwave range to improve the performance of insulation coatings based on polyvinyl chloride. It presents the results of studying the energy flow effects of microwave radiation on the evolution of the structure of polymer insulation materials during their processing. It is shown that the absorption capacity of polar polymers is significantly higher than that of nonpolar ones due to a significant dipole moment, because of which it is possible to achieve a deeper rearrangement of the polar polymer structure under microwave exposure. Unlike polyethylene, polyethylene terephthalate, and polypropylene, polyvinyl chloride (PVC) has a high absorption capacity for microwave radiation. This is explained by the fact that PVC is a polar polymer and its molecules are highly polarized, resulting in an increase in the strength of the insulation material based on PVC, a decrease in its water absorption, an increase in volumetric electrical resistance, and an increase in the glass transition temperature [9,10,11].

In the 2010s at Volgograd State Technical University, Kablov V.F. and Provotorova D.A. studied the modification of rubbers under microwave exposure. They found that, depending on the wavelength microwave exposure, it can influence certain types of chemical and physical bonds, which makes it possible to selectively carry out certain reactions. The processing of unsaturated rubbers by microwave radiation leads to an increase in the strength properties of rubbers based on ethylene propylene diene rubber by 20–30%, chloroprene rubber by 70–100%, and the acceleration of vulcanization by 10–15% [12,13].

The use of microwave technology makes it possible to reduce the depolymerization duration (15 times) and reduce energy consumption during the secondary processing (recycling) of the plastics used (plastic bottles made of polyethylene terephthalate) [14].

The microwave electromagnetic unit known to be used for technology intensifies the polymerization and depolymerization of thermosetting and thermoplastic polymers, where microwave radiation acts as a catalyst for chemical reactions, accelerating them by 5–200 times [15].

The mechanisms and principles of swelling various polymeric materials under microwave exposure have been studied by scientists in many countries around the world [16,17,18,19]. They demonstrated its promise for industrial implementation compared to traditional methods, which is the possibility of swelling without solvents, thus, reducing the processing time, improving energy efficiency and product quality. There are many active sites for the formation of pores, as well as the possibility of fine-tuning the heating energy to provide greater uniformity and additional control over the growth of pores, creating well-swollen, fine-pored, uniform foams.

This study allows us to show the advantages of obtaining heat-insulating materials under the action of microwave radiation, compared with traditional convective heating, as can be seen from the characteristics of their properties and macrostructure.

## 2. Theoretical Basis

Considering the positive experience of using microwave radiation to improve the properties of organic polymeric materials, a hypothesis has been proposed about the possibility of its use when manufacturing materials based on an inorganic polymer, i.e., liquid glass. The main goal of researching the technology of liquid glass thermal insulation materials using microwave radiation is to create highly efficient energy-saving methods for their manufacturing and processing, and to improve their performance properties by modifying the microwave electromagnetic field. The possibility of a significant reduction in material and energy costs causes special effects of electromagnetic microwave radiation, which can be both thermal and directly energy activating, although it is often difficult to separate them.

Due to the volumetric heating and the mechanism of the “non-thermal” effect of microwave radiation on the processed objects, the duration of their heating is significantly reduced, and the performance properties of products are greatly improved. Therefore, it seems relevant to study the mechanism of the effect of microwave exposure on the macrostructure, and physical and mechanical properties of thermal insulation materials based on the liquid glass, in order to create a new microwave technology for their manufacturing, which would ensure their energy-efficient processing and improve their performance properties.

## 3. Materials and Methods

The paper carries out a comparative assessment of the structure and properties of composite thermal insulation materials (TIM) manufactured under microwave exposure and traditional convective heating. The studies were carried out on a microwave unit with a standard operating frequency of 2.45 GHz and an output power of 300 W, which corresponds to a temperature of 55–60 °C; 500 W—a temperature of 100–110 °C; and 650 W—a temperature of 115–120 °C.

The liquid glass composition (LGC) used for manufacturing composite thermal insulation materials contains sodium liquid glass (LS) as the main component, zinc oxide and hemihydrate gypsum as modifiers of coagulation and crystallization, hydrogen peroxide as a foaming agent, and ethoxylated alkylphenol as a foam stabilizer. Non-swollen granules based on ZhS and zinc oxide are used as granular fillers. The manufacturing of thermal insulation materials has been carried out according to the technology given in [20,21,22]. The considered problems and presented solutions are also important for applications in transport systems [23,24,25,26].

Each study was conducted on at least five samples. The physical and mechanical characteristics of composite TIMs were determined in accordance with the current Ukrainian standards and international ISO standards using the methods given below. Before testing, the samples were conditioned for 24 h at a temperature of 20 ± 2 °C and a relative humidity of 50 ± 5%.

The middle material density shows the extension of the mass of the eye to the volume occupied by it, including the volume of the gas phase, which is given by the formula

(1) $$\rho =\frac{m}{V}$$

where *m* is the mass of the sample and *V* is the sample volume.

Water absorption was determined on rectangular samples with dimensions of 100 mm × 100 mm × 35 mm. The samples were dried to a constant mass at a temperature of 50–60 °C, then weighed to the nearest 0.01 g. The sample was immersed in water; the sample must be immersed in water for the first 3 h to half the thickness, and during the rest of the test time it must be completely immersed in water. After 24 h, the sample was removed from the water, and excess water was removed from its surface and weighed. The mass of water that spills onto the scale cup from the voids of the sample during weighing is included in the mass of the water-saturated sample. Water absorption (in %) was calculated according to the formula

(2) $$W_{n}=\frac{m_{3}-m_{1}}{m_{1}}100$$

where *m*_1_ is the mass of the sample before saturation with water and *m*_3_ is the mass of the sample after saturation with water.

The strength characteristics of the materials were determined on the P-5 testing machine, which allows determining the reading of the destructive load with an accuracy of at least 5 N. To determine the compressive strength, the sample must have the shape of a cube with an edge length equal to the thickness of the product. The sample is placed on the base plate of the press so that the compressive force is directed parallel to the vertical axis of the sample, and the axis of the sample passes through the centre of the base plate of the press. The load on the sample should increase uniformly without jerks at a speed of 10 mm/min. Sensors installed on the press measure the strength characteristics of the sample during the test. The compressive strength was calculated using the formula

(3) $$\sigma_{c}=\frac{P}{l\;\cdot \;b}$$

where *P* is the compressive load, *l* is the sample length, and *b* is the sample width.

When determining the bending strength, the sample should have the shape of a parallelepiped with dimensions of 250 mm × 10 mm × 10 mm. The sample is placed on two supports that have rounding at the joints. The distance between the axes of the supports should be 200 mm. The load on the sample is transmitted through a roller with a diameter of 10 mm and laid along the width of the sample at an equal distance from the supports. The destructive load is considered the load at which the destruction of the sample occurred. Bending strength was calculated using the formula

(4) $$\sigma_{u}=\frac{3P\;\cdot \;l}{2b\;\cdot \;h^{2}}$$

where *P* is destructive load during bending, *l* is the distance between the axes of the supports, *b* is the sample width, and *h* is the sample thickness. 

## 4. Results and Discussion

It should be noted that at a microwave power of 300 W and a convective heating temperature of 100 °C, swelling of the liquid glass composition does not take place. There is only drying of the material with a gradual loss of free and bound water. Therefore, experimental data for these swelling modes are not presented. The minimum temperature of convective heating for the LGC swelling is approximately 200 °C, while under microwave exposure this temperature is approximately 100 °C (radiation power 500 W).

Microwave exposure during the swelling of composite materials makes it possible to obtain materials with a more uniform structure and, as a result, higher performance properties compared to traditional convective heating.

**Figure 2:**
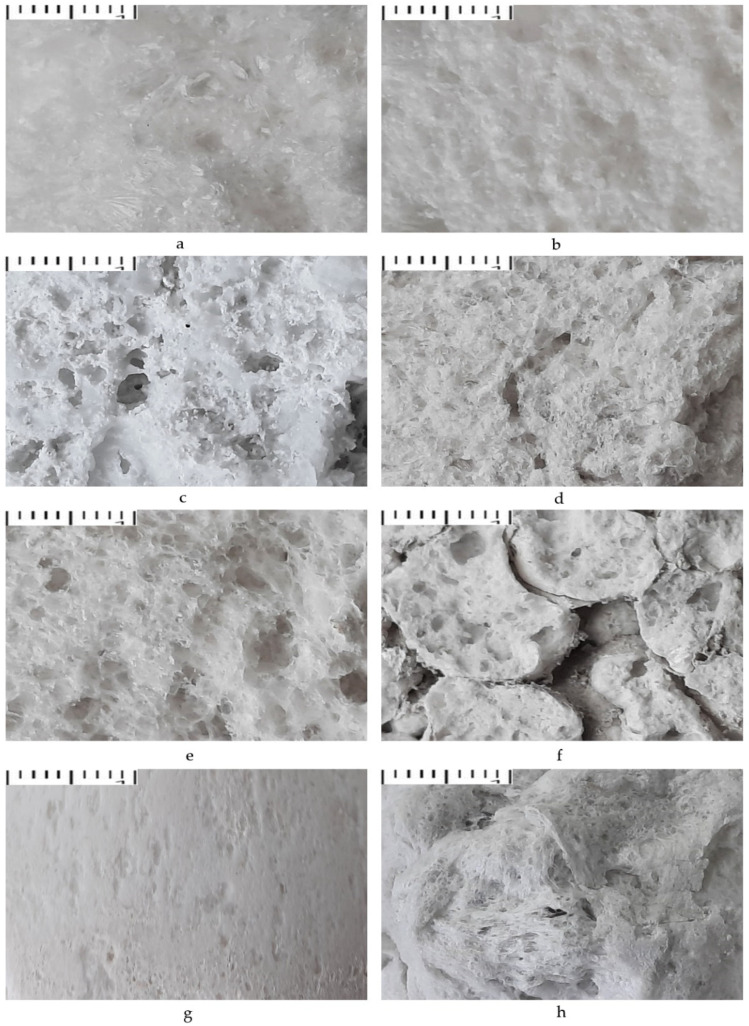
The porous structure of the composite material obtained under: (**a**) microwave exposure (power 500 W); (**b**) microwave exposure (power 650 W); (**c**) convective heating (T = 200 °C); (**d**) convective heating (T = 300 °C); (**e**) convective heating (T = 500 °C); (**f**) convective heating (T = 600 °C); (**g**) the surface of samples obtained by microwave heating (power 650W); (**h**) the surface of samples obtained by convective heating at T = 300–500 °C (magnification × 10 for all samples).

The porous structure of the materials (in section) obtained at different parameters of microwave and convective heating is shown in Figure 2.

From photographs a and b, the materials obtained under the action of microwave radiation have a more uniform finely porous structure and the granules do not separate from each other. The sample consists of gas-filled cells separated by the thinnest partitions; these partitions are not loose and porous, unlike materials obtained by convective heating, but are solid, smooth, and fused. On the surface of the sample under the action of microwave radiation, a compacted surface layer is formed, due to which the water absorption indicators decrease and the strength indicators increase. Materials obtained by convective heating at a temperature of 300–500 °C have a more heterogeneous structure (Figure 2d,e), especially in the surface layer (Figure 2h). Therefore, they are characterized by a predominantly open-pore structure. Materials obtained at a temperature of 600 °C have many through voids (Figure 2f). However, their strength is the highest due to the low swelling ratio. Materials obtained at a temperature of 200 °C consist of non-swollen granules and a non-uniformly swollen binder (Figure 2c), which indicates the impossibility of swelling composite materials at this temperature.

**Figure 3:**
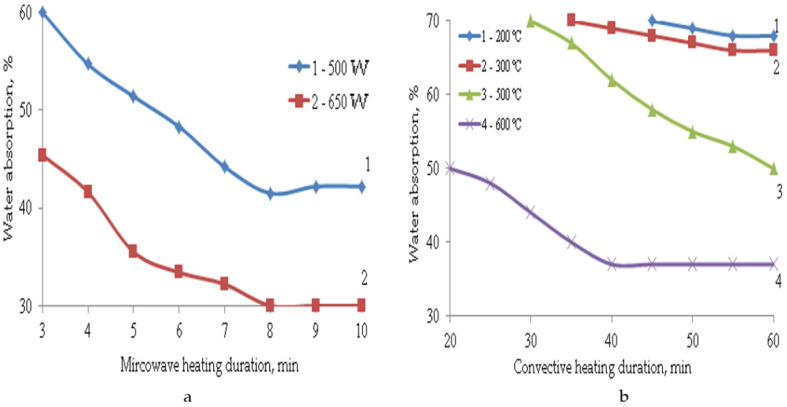
Dependence of water absorption on the parameters of microwave (**a**) and convective (**b**) heating.

**Figure 4:**
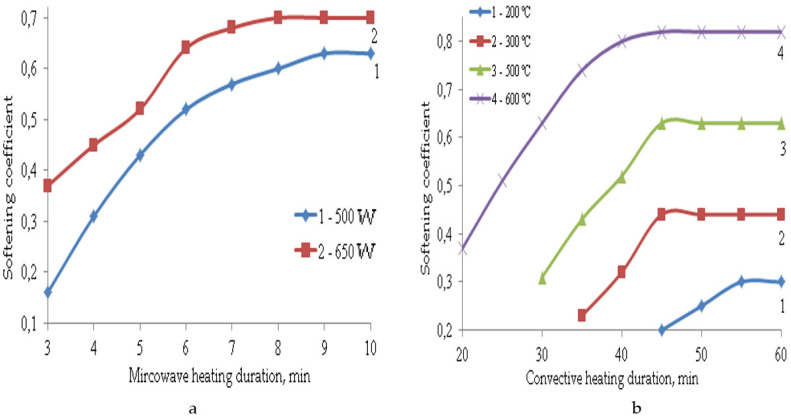
Dependence of the softening coefficient on the parameters of microwave (**a**) and convective (**b**) heating.

**Figure 5:**
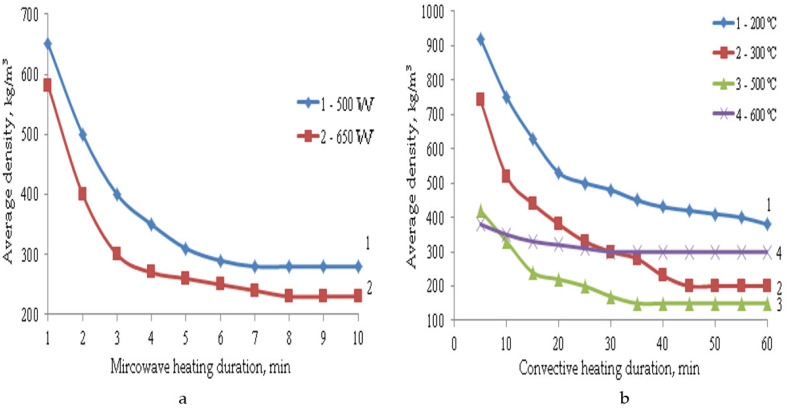
Dependence of the average density on the parameters of microwave (**a**) and convective (**b**) heating.

**Figure 6:**
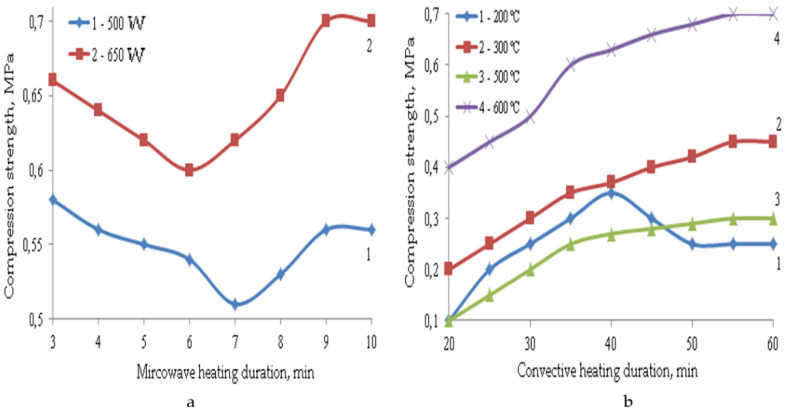
Dependence of compression strength on the parameters of microwave (**a**) and convective (**b**) heating.

**Figure 7:**
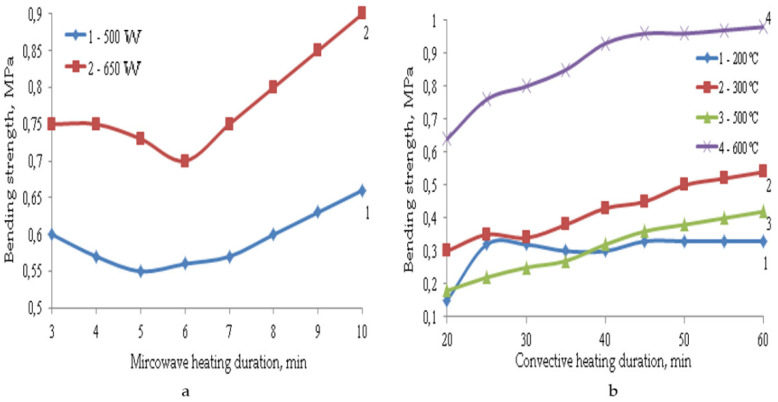
Dependence of bending strength on the parameters of microwave (**a**) and convective (**b**) heating.

The formation of such a porous structure of TIMs affects their performance properties. The main properties of composite materials depending on the parameters of microwave and convective heating are shown in Figure 3, Figure 4, Figure 5, Figure 6 and Figure 7.

Tests for water resistance and strength were carried out on samples with sufficient structural strength. Therefore, these do not include sample swelling and curing, which occurred within 1–2 min under microwave exposure and 5–15 min under convective heating.

Due to the formation of a compacted surface layer under microwave exposure and a predominantly closed-cell structure of TIM, the water absorption indices of such samples are 1.5–2 times lower than in the case of convective heating. Even at the highest temperature of 600 °C, water absorption is 37% after 45–60 min of heating, while when the samples are exposed to microwave radiation for 6-8 min, water absorption is only 30%. The samples obtained at a temperature of 200–500 °C have increased water absorption (50–70%) due to the formation of an inhomogeneous open-pore structure. Their softening coefficients are also too low at 0.3–0.6, so such materials are not waterproof. The most water-resistant materials are those obtained at a temperature of 600 °C. The value of their softening coefficient is 0.82. Materials obtained under microwave exposure at a power of 650 W are limitedly waterproof since their softening coefficient is 0.7. The softening coefficient was used to evaluate the water resistance of materials. It is the ratio of the strength of a material saturated with water to its strength in a dry state. Waterproof materials have a softening factor value greater than σ = 0.8. At σ = 0.7–0.8 the materials are limitedly waterproof. If the softening coefficient is less than 0.7, the materials are not waterproof. They are not recommended for use in structures and structures operating in conditions of high humidity.

Data in Figure 5 show that with an increase in temperature and duration of heat treatment, both in the case of microwave and convective heating, a decrease in the average density of the composite material is observed. Heating the LGC under microwave exposure contributes to a more complete release of chemically bound water and the formation of a highly porous material with low average density. Thus, the average density of the composite TIM obtained under microwave exposure with a power of 650 W is 230 kg/m^3^. The samples obtained at 300 °C under convective heating have a close value of the average density (200 kg/m^3^), but their physical and mechanical properties are much lower. If we study swelling at a temperature of 600 °C, it becomes clear that at this temperature the LGC curing is significantly activated, which makes it difficult for the material to swell, since sodium silicate crystallizes. As a result, the average density of such samples is two times lower than at a temperature of 500 °C, and is 300 kg/m^3^, while at a temperature of 500 °C it is only 150 kg/m^3^. However, with a decrease in the average density, there is also a decrease in the TIM strength.

Under convective heating with an increase in the heat treatment duration, the strength of composite materials increases. Even though swelling is already over after 30 min of heat treatment in most cases, the increase in strength continues further, and it reaches its constant value after an hour of heat treatment, which can be explained by the structure formation. Under the action of microwave radiation, the swelling process is completed after 8 min of heating. The decrease in the strength of materials at the initial stage can be explained by a decrease in their density during swelling. A further increase in strength is explained by the completion of structuring processes, which proceed more intensively with an increase in radiation power from 500 to 650 W. Under microwave exposure with an increase in radiation power from 500 to 650 W, the TIM strength changes insignificantly. This is due to the higher average density of the samples at the beginning of swelling, since it finishes after 8 min of heating. During convective heating, despite the high values of the average density of materials at the beginning of the process, their strength values are not high.

If we compare the strength of samples with a similar average density obtained at a microwave power of 650 W and at a convective heating temperature of 300 °C, it can be noted that the compression strength in the case of microwave radiation is 1.5 times higher, and the bending strength is 1.7 times higher, since microwave radiation eliminates the destructive effect of thermomechanical stresses on the material being processed. This happens because the temperature gradient in the sample is significantly reduced, thereby reducing internal stress. In addition, the duration of heat treatment is 5–6 times longer, and the process temperature is almost three times higher. The materials obtained at a temperature of 600 °C have the highest strength indicators, but the average density of such samples is 1.3 times higher. In addition, due to their heterogeneous structure, such samples have high thermal conductivity. Therefore, their coefficient of thermal conductivity is 0.07–0.08 W/m·K, and for the TIM obtained by microwave heating, this coefficient is 0.05–0.055 W/m·K.

Based on the conducted studies, it can be concluded that composite TIMs obtained under microwave exposure at a power of 650 W have the best set of performance properties. The required duration of such heat treatment is 8–10 min. The closest to them in terms of the swelling coefficient are materials obtained by convective heating at a temperature of 300 °C for 1 h. The comparative properties of such materials are given in Table 1.

Based on the above data, using microwave exposure, it is possible to obtain composite TIMs with a better set of performance properties related to modification of the liquid glass matrix due to the “non-thermal” effect of microwave radiation. In addition, volumetric microwave heating of all the LGC layers allows uniformly removing water without overheating in local areas. This prevents the formation of large voids in the sample structure, since microwave radiation acts directly on the water contained in the original LGC, while energy is not spent on heating the unit mould and walls. Due to the instantaneous effect of microwave exposure on the LGC, the rapid evaporation of free and bound water immediately leads to pore formation and the LGC curing, and these rates are well correlated with each other. Due to the formation of a predominantly closed-pore structure of the TIMs, the indicators of their water absorption and hygroscopicity are two times less important. The strength properties of composite materials obtained by microwave heating are 1.5–1.7 times higher than those obtained by convective heating, even though the average density of such samples is 1.3 times lower. This indicates the formation of the less defective three-dimensional structure of the LGC, whereas with convective heating, the curing of the LGC did not occur completely, and the binder was only dried.

## 5. Conclusions and Further Research

**Figure 8:**
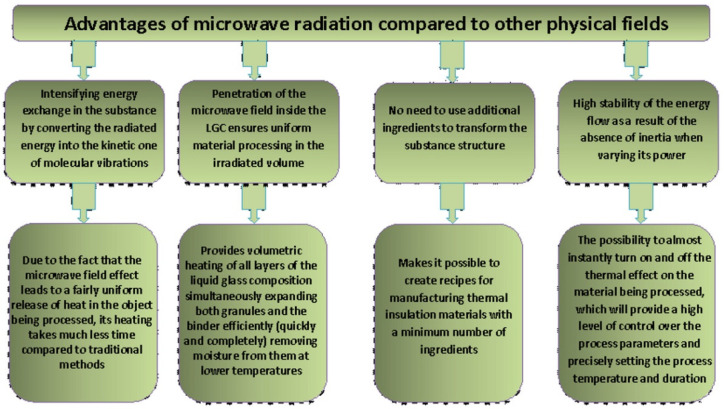
Advantages of microwave exposure for manufacturing TIM based on LG.

Thus, using microwave exposure during the swelling of composite materials makes it possible not only to obtain materials with a more uniform structure and higher performance properties, but also to reduce the swelling duration and temperature compared to traditional convective heating. The advantages of microwave processing are shown in Figure 8.

In the course of the conducted research, by a comparative assessment of the macrostructure and operational properties of composite heat-insulating materials based on an inorganic polymer (liquid glass), obtained under the action of microwave radiation and under convective heating, the prospects of using a microwave electromagnetic field in the production of new building materials, expanding their range, and improving quality and competitiveness can be achieved, which is a priority for the modernization and innovative development of the construction industry.

## Figures and Tables

**Table 1:** Properties of composite thermal insulation materials obtained by microwave and convective heating.

No.	Indicator Name	Indicator Value	
		Convective Heating	Microwave Heating
1	Average density, kg/m^3^	200–220	220–240
2	Humidity, %	1.6–1.7	1.4–1.5
3	Hygroscopicity, %	14–15	4.5–5
4	Water absorption, %	65–67	30–31
5	Total porosity, %	87–88	83–84
6	Content of closed pores, %	26–28	53–54
7	Softening factor	0.43–0.45	0.68–0.7
8	Compression strength, MPa	0.4–0.5	0.6–0.7
9	Bending strength, Mpa	0.5–0.6	0.8–0.9
10	Thermal conductivity coefficient, W/m·K	0.07–0.08	0.05–0.055

## Data Availability

Not applicable.
